# Genotypic and Phenotypic Diversity as a Function of CRISPR-Directed Gene Knock-Out of *NRF2* in Pancreatic Adenocarcinoma Cells, a Feasibility Study

**DOI:** 10.3390/biom16060828

**Published:** 2026-06-03

**Authors:** London P. McGill, Kelly H. Banas, Gregory Tiesi, Eric B. Kmiec

**Affiliations:** 1 Gene Editing Institute, ChristianaCare, Newark, DE 19713, USA; 2 Department of Medical and Molecular Sciences, University of Delaware, Newark, DE 19713, USA; 3 Division of Surgical Oncology, HMH Jersey Shore University Medical Center, Neptune, NJ 07753, USA

**Keywords:** CRISPR, gene ablation, drug resistance, NRF2

## Abstract

Pancreatic ductal adenocarcinoma (PDAC) presents unique treatment challenges, often due to the development of anti-cancer drug resistance. Previously, we demonstrated that CRISPR-directed gene ablation disabled the master regulator gene *NRF2*, a transcription factor known to control drug resistance in squamous cell carcinoma tumor cells, and restored chemosensitivity. In this short study, we evaluated a broad range of CRISPR/Cas9 molecules for their capacity to elicit similar responses in PDAC cells. Synthetic single guide RNAs (sgRNAs) were designed to target multiple functional domains encoded by NRF2. These molecules were delivered to cells via nucleofection, with outcomes analyzed by genotypic, phenotypic, and functional assays. We observed targeting efficiencies ranging from 25% to 100% with a high level of random insertions and deletions (indels). sgRNAs targeting exons 2, 3 and 4 demonstrated a high degree of genotypic, phenotypic and functional outcomes. Targeted disruption of exons 3 and 4 reveals significant loss of cell viability while overcoming drug resistance through the restoration of sensitivity to gemcitabine (>1.75 μM). Our study identifies domain-specific sites within NRF2 that, when disabled, restore sensitivity to gemcitabine, potentiating a more in-depth analysis of this novel augmentative therapeutic approach.

## 1. Introduction

Pancreatic ductal adenocarcinoma (PDAC) remains one of the most challenging cancers to treat. Despite improvements in earlier-stage detection [1], most patients will still require some form of systemic therapy due to (1) high incidence of metastatic disease at presentation, (2) significant risk of recurrence in resected disease or (3) advanced local involvement, making surgery unfavorable or even impossible at diagnosis [2,3,4,5,6]. Complete pathologic response is exceedingly rare, and, over time, many patients develop resistance to therapy [7,8,9,10,11,12,13,14,15]. Thus, the five-year survival remains dismal at just ~10%, in resectable disease, and <1% for patients diagnosed at stage IV [3,5,6], making it the 3rd leading cause of cancer related death in women and the 4th leading cause of cancer related deaths in men in the United States at an average of 8% of cancer related deaths across both genders [16].

We are advancing a novel strategy to reduce treatment resistance in solid tumors by CRISPR (clustered regularly interspaced palindromic repeats)-directed gene knockout. A CRISPR approach, although much more precise, is not dissimilar to that of standard cancer therapy. Anticancer drugs also induce double-strand breaks, which trigger a cascade of repair events, guided by the DNA damage response pathway, to rejoin the chromosome [17,18,19]. In many cases, breaks are repaired by non-homologous end joining [17,20,21,22,23], which is unfaithful, causing loss or gain of DNA nucleotides and inevitably altering the reading frame, leading to disruption of gene function. CRISPR drugs act only at the level of the gene [24,25], rendering them unsusceptible to conformational changes in biomarkers or proteins opposite that of cell or immunotherapies. The CRISPR complex also acts with remarkable precision to generate double-stranded breaks, which overcome the genetic block preventing other cancer therapies from working effectively.

As a first step in the long clinical journey, we begin to explore the feasibility of CRISPR-directed gene editing to augment gemcitabine response in treatment-resistant pancreatic cancer cells. We build on previous work, which demonstrated that gene knock-out could be used to disable gene function involved in treatment resistance [26,27,28,29]; in particular, the Nuclear Factor Erythroid 2-Related Factor (NRF2, encoded by the *NFE2L2* gene) potentiates much of the drug resistance in squamous cell lung cancer and head and neck cancer [27,29]. The encoded protein, NRF2, is a master regulator transcription factor that impacts the effectiveness of standard cancer care by activating cellular pathways that lead to treatment resistance [30,31,32,33,34,35,36,37,38,39,40]. In the case of treatment resistance in PDAC, NRF2 has been implicated in promoting resistance to gemcitabine through activation of antioxidant responses to reactive oxygen species (ROS) and through upregulation of protective genes like NQO1, HMOX1 and GCLC [36,37]. Our long-term goal is to reduce the amount of systemic therapy required to promote cell death in drug-resistant pancreatic tumor cells by employing CRISPR-directed gene knock-out to disrupt the function of NRF2. We sought to identify sites within NRF2 that are most amenable to genetic disruption, enabling the restoration of sensitivity to gemcitabine. The results of our experiments herein identify several CRISPR/Cas complexes that display high activity in PANC-1 and Mia-Paca-2 cells, thereby establishing a framework upon which we can further develop a complementary genetic approach to the treatment of pancreatic cancer.

## 2. Materials and Methods

### 2.1. Cell Line and Culture Conditions

Human pancreatic ductal adenocarcinoma cells (PANC-1 and Mia-Paca-2) were purchased from ATCC (Manassas, VA, USA). Cells were thawed according to the manufacturer’s protocol. PANC-1 cells were cultured in Dulbecco’s Modified Eagle’s Medium (DMEM) (ATCC, Manassas, VA, USA) supplemented with 10% FBS. Mia-Paca-2 cells were cultured in Dulbecco’s Modified Eagle’s Medium (DMEM) supplemented with 10% FBS and 5% horse serum. All cells were grown at 37 °C in 5% CO_2_.

### 2.2. CRISPR/Cas9 Design

The *NRF2* gene-coding sequence was downloaded into a SnapGene file and then the Synthego (Menlo Park, CA, USA) CRISPR design tool was used to select sequences across the *NRF2* gene in exon 2 (sgRNA1: 5′-TGGATTTGATTGACATACTT-3′, sgRNA3: 5′-TGGAGGCAAGATATAGATCT-3′, sgRNA2: 5′-TAGTTGTAACTGAGCGAAAA-3′), exon 3 (sgRNA7: 5′-AGCATCTGATTTGGGAATGT-3′, sgRNA6: 5′-AAGTACAAAGCATCTGATTT-3′, sgRNA5: 5′-CCTCATTGTCATCTACAAAC-3′), exon 4 (sgRNA76: 5′-TCACTTGTTCCTGATATTCC-3′, sgRNA83: 5′-GTAGCCCCTGTTGATTTAG-3′), and exon 5 (sgRNA94: 5′-TGAGTTCACTGTCAACTGGT-3′, sgRNA107: 5′-AGACAAACATTCAAGCCGCT-3′). The sequences were uploaded into the SnapGene file, and ten gRNA sequences were selected for testing. Synthetic single sgRNAs were ordered from Synthego (Menlo Park, CA, USA) using the gRNA designs. SpCas9 2NLS Nuclease (1000 pmol) was also ordered from Synthego (Menlo Park, CA, USA).

### 2.3. Nucleofection Transfection

Three million cells were seeded into a 75 cm^2^ tissue culture flask 24 h prior to transfection and allowed to reach 60–80% confluency. On the day of transfection, RNP was complexed using sgRNA and spCas9 at a 5:1 (250:50 pmol) ratio and left to incubate at room temperature for 15 min. While RNP was incubating, cells were harvested, and one million cells were placed into a 1.5 mL tube. The cells were spun down at 300× *g* for 5 min, and the media were aspirated from the tube before the cells were resuspended in 1 mL PBS. The cells were then spun down again, and PBS was aspirated from the tube before cells were resuspended in 100 µL Lonza SE solution (Lonza, Walkersville, MD, USA). 5 µL of RNP complex was added to resuspended cells, and cells were nucleofected using the Lonza program EO-137 [1]. Cells were resuspended in 500 µL pre-adapted media and left in the incubator for 10 min. Cells were then plated in a T25 flask and left to recover for 72 h.

### 2.4. Primer Design

Primers were designed for each exon of interest using Primer3Plus and evaluated for self-annealing. Primers were then ordered from IDT (Coralville, IA, USA). Exon 2 FWD: 5′-CACCATCAACAGTGGCATAATGTGAA-3′, REV: 5′-AACTCAGGTTAGGTACTGAACTCATCA-3′, Exon 3 FWD: 5′-GTGGTCTAGTTCAAATTGTGC-3′, REV: 5′-GGTTATGCTGTCCATGTTTC-3′, Exon 4 FWD: 5′-GTAGTGGTGCCTTAGAGCTTACTCATCC-3′, REV: 5′-CTAGCATGGGCAGTACTCATGACTAAG-3′, Exon 5 FWD1: 5′-GCCTGAAGATAATGTGGGTA-3′, REV1: 5′-CCTCCAAGCGGCTTGAATGTTT-3′, FWD2: 5′-AACCCTTGTCACCATCTCAGGG-3′, REV2: 5′-TCTTACCCCTCCTACGTATATC-3′.

### 2.5. Sanger Sequencing and Gene Editing Analysis

Genomic DNA was extracted from harvested cells using the Lucigen QuickExtract™ DNA Extraction Solution (LGC Biosearch Technologies, Middleton, WI, USA; Cat. QE09050). Amplicons were designed to encompass the CRISPR target site for each exon within the *NRF2* gene: exon 2 (530 bp), exon 3 (517 bp), exon 4 (402 bp), exon 5.1 (857 bp), and exon 5.2 (306 bp). Amplification was done using Phusion™ High-Fidelity Master Mix with HF Buffer (New England Biolabs, Ipswich, MA, USA; cat. M0531L). Post-amplification purification was done using the QIAquick PCR Purification Kit (QIAGEN, Hilden, Germany; Cat. 28106). Samples were prepared for Sanger sequencing using BigDye™ Terminator v3.1 Cycle Sequencing Kit (Applied Biosystems, Thermo Fisher Scientific, Waltham, MA, USA; Cat. 4337455); 20–30 ng of purified genomic DNA was amplified using primer sets from the initial reaction. Once the BigDye™ Terminator run was complete, samples were cleaned using the BigDye XTerminator™ Purification Kit (Thermo Fisher, Cat. 4376486) and placed into the SeqStudio platform for analysis and readout. Readout was analyzed post-sequencing using DECODR™ v 3.0 software (ChristianaCare Gene Editing Institute, Newark, DE, USA).

### 2.6. Cell Viability

Wildtype and transfected PANC-1 cells were plated in 24-well plates at 50,000 cells per well and placed in an incubator for 24 h. The cells were then treated with either gemcitabine (Selleckchem, Houston, TX, USA; Cat. S1714) or clinical-grade gemcitabine (a gift from ChristianaCare Pharmacy, Newark, DE, USA). Cells were treated at one of the following concentrations: 0 µM, 1.75 µM, 2.5 µM, 5 µM. Cells remained in gemcitabine for 72 h before being evaluated using CellTiter-Glo 2.0 Cell Viability Assay (Promega, Madison, WI, USA; Cat. G9241). A 1:1 cell suspension made from each biological replicate was loaded into a white 96-well plate in triplicate experimental replicates. The plate was then covered with foil and placed on an orbital shaker for 2 min. The plate was then left to sit at room temperature for 10 min before luminescence was measured using an Infinite 2000 PRO microplate reader (Tecan, Männedorf, Switzerland). Normalized data were analyzed in GraphPad Prism 10, nonlinear regression analysis was performed, and the CC50 value was derived from the fitted curve.

### 2.7. Western Blot Protein Analysis

PANC-1 cells were collected using a standard RIPA lysis buffer containing a protease inhibitor cocktail (Pierce, Rockford, IL, USA) and incubated on ice for 30 min, with vortexing done every 10 min. Samples were spun at 14,000× *g* in a 4 °C centrifuge for 15 min. Total protein concentration of collected supernatant was then determined using the BCA Protein Assay kit (Pierce, Rockford, IL, USA). 20 µg of protein was then mixed 3:1 with Laemmli buffer, 5% Beta-mercaptoethanol (BioRad, Hercules, CA, USA) and boiled at 95 °C for 10 min and then underwent SDS-PAGE on a 10% Mini-PROTEAN TGX Stain-free Protein Gel (BioRad, Cat. 4568033) for 90 min at 100 V. The gel was then transferred to a nitrocellulose membrane using Trans-Blot Turbo Transfer Systems (BioRad, Hercules, CA, USA) with Trans-Blot Turbo RTA Mini 0.2 µm Nitrocellulose Transfer Kit (BioRad Cat. 1704158), mixed molecular weight program (1.3 A, 25 V, 10 min). The blot was placed in 5% milk and blocked at room temperature on the shaker for 2 h and stained with anti-NRF2 1:1000 (Abcam, Cambridge, MA, USA; ab62352) and anti-Gapdh 1:5000 (Cell Signaling Technology, Danvers, MA, USA; 97166) overnight at 4 °C on the shaker. Blot was washed 3X in TBS-T, 10 min per wash, and stained with secondary antibody conjugate HRP 1:10,000 (Abcam, ab205718 or Thermofisher, PI31430). Pierce Fempto Western blotting substrate (Pierce) was used to image blots.

### 2.8. Statistical Analysis

An ordinary one-way ANOVA with Dunnett’s multiple-comparison test was performed on the dose curve; each population was analyzed within each dosing group and compared to the unedited population for each. This analysis was done using GraphPad Prism 10, showing a statistically significant difference. Statistical significance is reported in the graph ((C) of the second figure in Section 3), and *p*-values are reported in the Section 3. Error bars shown were calculated using CV from experimental replicates. For editing efficiency ((A) of the second figure in Section 3) and frameshift analysis ((B) of the second figure in Section 3), the median value for all biological replicates shown was calculated and displayed on charts.

## 3. Results

**Figure 1:**
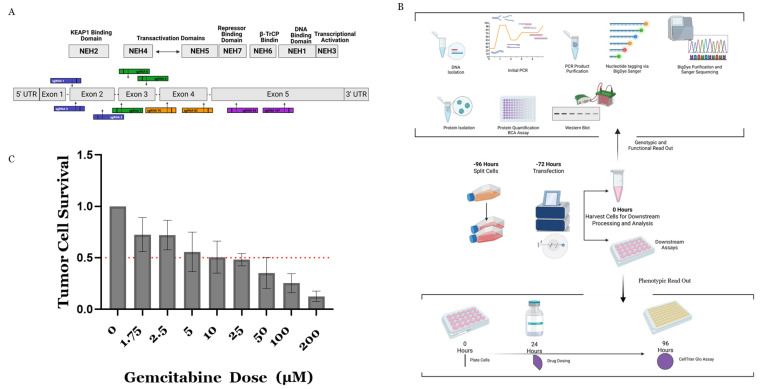
(**A**). Structural Domains of NRF2 and CRISPR Design and guide RNAs (sgRNA) with target region indicated. Exon 2 contains the KEAP1 binding domain and its protein domain Neh2, Exons 3 and 4 encode the transactivation domain with two protein domains, Neh4 and Neh5, respectively, and Exon 5 contains multiple functional domains with their respective protein domains. SgRNAs were designed to target within these functional and protein domains. (**B**). Experimental workflow. PANC-1 and Mia-Paca-2 cells undergo transfection with sgRNAs, and isolated genomic DNA is sequenced for editing efficiency, indel population analyses and chemosensitivity. Phenotypic, functional, and genotypic responses are recorded. (**C**). Phenotypic response of unedited PANC-1 cells, establishing the dose curve for later experiments (created in Biorender.com).

NRF2 is a transcription factor that regulates downstream genes responsible for antioxidant response pathways, which can promote chemotherapy resistance, tumorigenesis, and many other key functions in cancer [30,33,34,35,41]. Figure 1A displays the structure of the *NRF2* gene, which is divided into five exons encoding different functional protein domains. Ten synthetic single guide RNAs (sgRNAs) were designed to disrupt NRF2 across four exons: exon 2 (sgRNA 1, 2, 3), exon 3 (sgRNA 5, 6, 7), exon 4 (sgRNA 76, 83), and exon 5 (sgRNA 94, 107). Guide RNA activity was tested in PANC-1 cells, an epithelial carcinoma line harvested from a 56-year-old patient with pancreatic ductal adenocarcinoma [42], well studied and considered to be one of the primary cell models for pancreatic cancer. This cell line is also known to be highly resistant to gemcitabine, a first-line chemotherapy treatment in pancreatic cancer [43].

First, we identified the dose curve of PANC-1 unedited cells in response to gemcitabine required to achieve approximately 50% cell viability using the CellTiter 2.0™ assay. (Figure 1C). Once we determined this dose (5 µM), two more doses between (0 µM) control and (5 µM) were selected so that the cell viability effect could be evaluated in cultured cells without losing the entire population.

**Figure 2:**
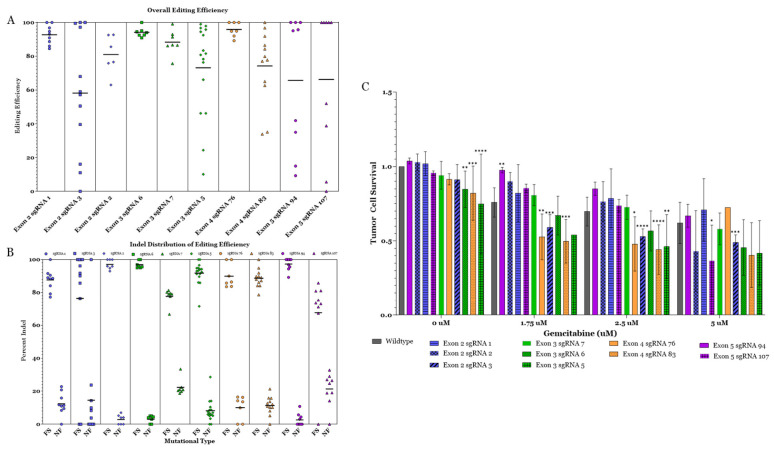
Genotypic and phenotypic response to CRISPR-directed editing of the *NRF2* gene in PANC-1 cells. (**A**). Overall editing efficiency for each sgRNA: sgRNA 2 (*n* = 6), sgRNA 3 (*n* = 12), sgRNA 1, 94, 107 (*n* = 9), sgRNA 6, 7 (*n* = 7), sgRNA 5 (*n* = 16), sgRNA 76 (*n* = 7), sgRNA 83 (*n* = 12). Asterisks above bars indicate statistical significance as follows: *: *p* ≤ 0.05, **: *p* ≤ 0.01, ***: *p* ≤ 0.001, ****: *p* ≤ 0.001. (**B**). Indel distribution of the edited population. Frameshifting versus non-frameshifting populations. (**C**). Dose curve capturing the response of the nontargeted control compared to targeted PANC-1 cells (only biological replicates with 60% editing efficiency or higher were plated and dosed) to gemcitabine exposure (created in GraphPad Prism 10).

Following the experimental workflow presented in Figure 1B, sgRNAs and Cas9 protein were introduced into PANC-1 cells as Ribonucleoprotein Complexes (RNPs) via nucleofection. Seventy-two hours afterward, the transfected cells were harvested, processed for Sanger sequencing and analyzed using DECODR [44] to reveal a detailed insertion and deletion (indel) profile ((A) of the third figure in Section 3). All sgRNAs were effective in promoting gene editing with a range of mutational outcomes (Figure 2A). While the same amount of RNP complex was introduced into the same cell line at the same time, each locus generates a spectrum of editing efficiencies [20,21,22]. The efficacy of gene editing is often taken as a core metric, predictive of the efficiency of phenotypic outcome. While the current trend is to examine the effectiveness of gene editing based solely on this metric, it can be deceiving. Hence, we believe that altered functional outcomes are more aptly dependent on the spectrum of insertions and deletions catalyzed by each CRISPR/Cas complex.

**Figure 3:**
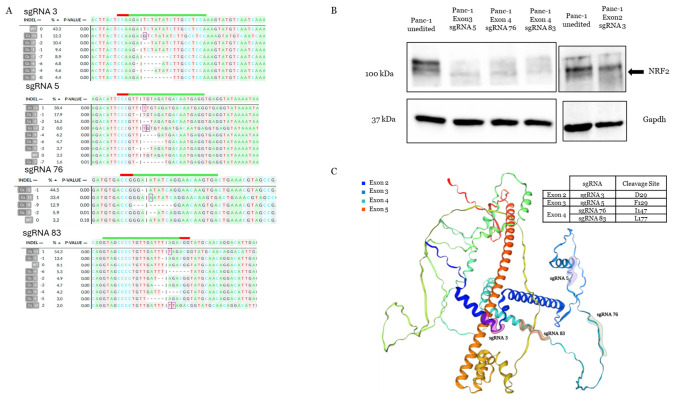
Phenotypic response of PANC-1 cells. Edited populations using sgRNAs of interest. (**A**) DECODR readout of Sanger sequencing showing indel spectrum of bulk populations for sgRNA 3, 5, 76, and 83. (**B**) Western blot showing protein levels of unedited cells compared to edited cells from populations (sgRNA 3, 5, 76, 83). (**C**) Protein structure of NRF2 conceptualized using SWISS-MODEL [48], with amino acid sites highlighted that correlate to the DNA target sites of each CRISPR sgRNA. Original Western Blot images can be found in Supplementary Materials.

We know that frameshift mutations (+1, +2, −1, −2, etc.) are likely to change the open reading frame (ORF) of a coding region, potentially leading to higher levels of functional and phenotypic disruption. We define frameshift as any indel occurring in a sum not divisible by three, and we define non-frameshift as any indel divisible by three. This classical view is because we define a codon as a set of three nucleotides that encode for one amino acid. While the diversity of editing efficiencies has been reported [20,21,22], most studies do not determine or report effective editing efficiencies, those that will likely generate the desired functional alteration. Thus, we proceeded to analyze the indel footprints (Figure 2A,B and Figure 3A) created by each of the 10 sgRNAs used in this study. The indel spectrums reveal a significant level of frameshift mutations, potentiating impactful functional knockout downstream. Interestingly, across the spectrum of overall editing efficiencies per sgRNA (exon 2: 58–96%, exon 3: 76–98%, exon 4: 76–98%, exon 5: 65% across all sgRNAs), the ratio of frameshift to non-frameshift remains approximately the same (76–96% frameshift, 4–24% non-frameshift), yet phenotypic response varies across sites.

Once we established a sufficient level of frame shift mutations being created through the action of the sgRNAs, we proceeded to analyze functional disruption of NRF2 through analysis of the restoration of chemosensitivity to gemcitabine. Cells were transfected and then exposed to gemcitabine. Unedited PANC-1 cells and PANC-1 cells treated with the appropriate sgRNA and gemcitabine exposure were compared (Figure 2C); these data were used to determine the sgRNA that had the largest impact on chemosensitivity restoration. These results are normalized and compared to unedited, untreated parental cells as displayed in Figure 2C. The data from each sgRNA is sorted by descending cell viability under no treatment (0 µM). As previously observed, non-targeted wildtype cells remain resistant to treatment with gemcitabine. Additionally, several cell populations targeted with sgRNAs against exon 2, exon 3 or exon 5 exhibit enhanced or maintained resistance to gemcitabine. Notably, exon 2 sgRNA 1 and sgRNA 2, exon 3 sgRNA 7, and exon 5 sgRNA 94 show a slight enhancement in resistance at 1.75 µM, 2.5 µM, and 5 µM of gemcitabine compared to the control, with each sgRNA having statistically significant increases in resistance at varying doses (Figure 2C). As a general observation, the overall response to gemcitabine from targeting up and down the *NRF2* gene reveals that exon 2 and exon 5 may enhance resistance, while targeting exon 3 and exon 4 may enhance sensitivity.

It has been shown that loss of NRF2 alone impairs cell viability [26,27,28,29,45,46], here we replicate these findings, observing modest cell viability loss with editing alone from sgRNA 3, 5, 76, and 83 (0 µM), with sgRNAs 5 and 83 showing statistically significant viability loss (*p* < 0.0001, *p* = 0.0001), and sgRNA 5 having the lowest viability of 80%. However, when coupled to gemcitabine, cells targeted with sgRNAs 3, 5, 76 and 83 respectively reveal enhanced sensitivity at 1.75 µM (*p* = 0.0009, *p*= 0.4862 (ns), *p* = 0.0042, *p* = 0.0009), 2.5 µM (*p* < 0.0001, *p* = 0.0059, *p* = 0.0142, *p* < 0.0001) and 5 µM (*p* = 0.0004, *p* = 0.2865 (ns), *p* = 0.9999 (ns), *p* = 0.1428 (ns)) when compared to the unedited control (Figure 2C). Interestingly, disruption of exon 2 with sgRNA 3, but not sgRNA 1 or 2, significantly enhances sensitivity to gemcitabine. At a concentration of 5 µM, the edited cell population achieves ~50% viability loss; in comparison, to achieve ~50% viability loss in unedited cells, the concentration of the drug must approach 10–25 µM (Figure 1C). SgRNA 3 appears to be an excellent candidate to explore in greater detail as an effective CRISPR molecule to support the killing of pancreatic cancer cells by gemcitabine, in addition to sgRNA 5 and sgRNA 83.

We profiled the genotypic composition of PANC-1 cell populations treated with sgRNA 3, 5, 76 and 83, respectively, using the bioinformatics deconvolution program, DECODR, presented in Figure 3A. These profiles reveal that significant genetic disruption has occurred, most likely accounting for the functional impact. The disruption in protein expression was also evaluated and displayed in Figure 3B. The Western blot shows a significant diminution in NRF2 protein levels 72 h after RNP nucleofection, reflecting a successful genetic knockout of NRF2.

While functional knockout of NRF2 through genetic disruption has been established in other cancer models [26,46], it is important to demonstrate utility in a second pancreatic cancer cell line, Mia-Paca-2, an epithelial carcinoma line harvested from a 65-year-old patient with pancreatic ductal adenocarcinoma. Although this line is derived from the same adenocarcinoma background, they are not reported to be resistant to gemcitabine [36,47]. Using the same experimental design, we targeted Mia-Paca-2 cells with gRNA 5, sgRNA 76 and sgRNA 83. Figure S1 illustrates the indel profiles and summary of overall editing efficiency in Mia-Paca-2 cells, as well as the reduction in NRF2 protein analyzed by Western blot. Gene editing activity remains high with significant activity from each respective guide RNA, and as expected, NRF2 protein levels are substantially reduced 72 h after RNP nucleofection. Our genotypic and phenotypic results suggest that the outcomes of NRF2 disruption are consistent in several pancreatic tumor cell lines.

## 4. Discussion

This study aimed to identify and characterize several CRISPR molecules that would effectively disrupt the function of NRF2 within the pancreatic ductal adenocarcinoma cells. NRF2 acts as a master regulator transcription factor, acting on downstream genes that activate therapeutic resistance in PDAC [30,31,32,33,34]. Identifying CRISPR molecules that partially ablate key functional domains within the gene lays the mechanistic foundation, facilitating work towards our long-term goal of developing an augmentative therapy for PDAC using gene knock-out.

The data shown in this manuscript highlight the importance of screening multiple sgRNAs within a single genetic target, show the importance of targeting within functional domains, and reveal that sgRNAs with cut sites near one another can provide vastly different outcomes. This study was focused on analyzing editing outcomes in treatment-resistant cells (PANC-1) and establishing the phenotypic outcome from targeting within different functional domains. An alternative cell line confirmed CRISPR activity at similar sites and across multiple PDAC backgrounds.

*NRF2* gene ablation as a therapeutic course to overcome cancer drug resistance in PDAC is supported by previous work in both squamous cell carcinomas of the lung [26,28,29] and head and neck cancer [27], where NRF2 disruption was shown to increase sensitivity to anticancer drugs. NRF2 is a transcription factor with a well-documented role in oncogenesis, implicated in the regulation of multiple genes that play a defensive role via antioxidant action by acting on the promoter regions of many downstream genes [30,31,32,33,34,35,49], including in PDAC [30,31,33,34,37,40]. Mouse studies demonstrate that constitutive activation of NRF2 protects pancreatic beta cells from oxidative damage and leads to more aggressive disease with increased metastatic potential [50]. NRF2 activation has also been shown to guard premalignant pancreatic cells from injury, facilitating tumor growth [30,51]. By targeting a transcription factor involved in various aspects of cancer survival, including drug resistance, genetic knockout has the potential to impact multiple oncogenic pathways.

While simply evaluating genetic knockout at the level of the DNA is a scientifically sound objective, it is the disruption of protein function that has the greatest impact. So, in the present manuscript, we not only measure overall gene editing efficiency, but we also correlate indel profiles, the percentages of frameshift mutations created by each guide RNA, with phenotypic response. This metric is important in evaluating successful gene knock-out in human cells, where the aim is to disrupt oncogenic pathways and tumor cell function. In our studies, most sgRNAs create frameshift mutations and a significant reduction in protein expression, readily visible by Western blot analyses. Importantly, we were able to demonstrate functional impact by restoring chemosensitivity to gemcitabine in PANC-1 cells, a third metric that demonstrates a tangible impact on function. NRF2 influences multiple cellular processes beyond direct ROS detoxification that can plausibly modulate gemcitabine response. Furthermore, studies have shown that altering NRF activity via therapeutics can affect gemcitabine sensitivity. Investigators in one study [40] demonstrated that inhibiting Nrf2 via Digoxin, combined with gemcitabine, led to increased cell death/sensitivity in a pancreatic cancer model.

One experimental goal was to identify a candidate CRISPR/Cas molecule that demonstrated sufficient gene editing efficiency, created an effective indel profile, reduced NRF2 protein expression and ultimately restored significant chemosensitivity to gemcitabine. We believe that we have met this objective, and interestingly, the optimal sgRNA in the Panc1 cells is one that targets exon 2. We had previously identified a tumor-specific sgRNA CRISPR/Cas complex that was effective in the genetic knockout of NRF2 in squamous cell carcinoma of the lung [26,27,28,29]. We potentiated that discovery by following with detailed molecular analysis and ultimately animal studies; this molecule is now in clinical development. Of the other three sgRNAs analyzed in this manuscript, sgRNA 3 and 76 are known and have been published in our previous work, and sgRNA 5 is new to this model system. Their efficiency in the pancreatic cancer cell model system, to produce the desired molecular and functional outcomes, shows the unique ability of CRISPR to reliably function across heterogeneous backgrounds and still produce similar outcomes.

Here, the discovery of the highly active sgRNA 3 CRISPR/Cas complex is most intriguing. It exhibits a broad range of gene editing activities in pancreatic tumor cell populations, but the distribution of frame shift and non-frame shift approximates other sgRNA activities. Our results provide a valuable lesson to workers in the field who do not carry out benchtop research to predict the impact of editing specific sites within the targeted gene. The dynamic nature of the chromosome and the complex interactions with molecular pathways in each cell type can skew results; for this reason, direct analyses by screening need to be carried out. Sometimes predicted genotype does not reflect phenotype, as we demonstrate in this manuscript with both sgRNA 1 and sgRNA 3 having high editing efficiency and sitting three bases apart, yet sgRNA1 shows statistically significant viability gain while sgRNA 3 shows statistically significant viability loss.

There is no doubt that sgRNA 3 is the most effective in the restoration of gemcitabine, even with a less active genotypic readout. There are several possibilities for this apparent contradiction. First, transcription of the *NRF2* gene can produce isoforms that can escape detection by Western blot analysis and still support at least some partial activation of downstream genes. In subsequent studies, we will evaluate isoform production in edited cells. By targeting an exon in the upstream region of any gene, there is an increased probability that all isoforms will be disabled. Second, previously, we have determined that targeting NRF2 within certain exons can lead to exon skipping, which by its very definition can produce active isoforms [26,29]; we will evaluate exon skipping in detail in the next round of studies. Perhaps neither of these two events takes place in cells in which the *NRF2* gene has been disabled by sgRNA 3.

## 5. Conclusions

In conclusion, we have started the process of evaluating CRISPR-directed gene ablation as a supplemental therapy for adenocarcinoma of the pancreas. As a first step, we demonstrated that CRISPR molecules can disable the function of NRF2 more efficiently than others. While this discovery is not novel, it provides a teachable moment for those working in the arena of genetic medicine. Not every CRISPR targeting site is created equal, and oftentimes disruption of a particular exon may result in insufficient impact on the desired biochemical or genetic readout. The next step is to develop clonal isolation of the targeted population of cells to determine what allelic composition facilitates the greatest amount of functional disruption. Once we understand that, and the percentages of those disrupted alleles present in any targeted population, we should be able to develop a hypothesis as to whether this approach is feasible. We will continue this line of investigation by conducting detailed molecular and functional analysis not in general cell populations, but in clonal cell populations, where stable, confirmed allelic distribution gives us the opportunity to establish structure/function relationships.

## Data Availability

The original contributions presented in this study are included in the article/supplementary material. Further inquiries can be directed to the corresponding author.

## Supplementary Materials

The following supporting information can be downloaded at: https://www.mdpi.com/article/10.3390/biom16060828/s1, Figure S1: Genotypic and phenotypic response of NRF2 targeting in Mia-Paca-2 cells; Figure S2: Original western blots PANC-1 cells; Figure S3: Original western blots PANC-1 cells; Figure S4: Original western blots Mia-Paca-2 cells; Figure S5. Densitometry calculations.

## Abbreviations

The following abbreviations are used in this manuscript:

CRISPR	clustered regularly interspaced palindromic repeats
PDAC	Pancreatic ductal adenocarcinoma
sgRNA	Synthetic single-guide RNAs
RNP	Ribonucleoprotein
indel	random insertions and deletions
