# Supplementary material for: Genotypic and Phenotypic Diversity as a Function of CRISPR-Directed Gene Knock-Out of NRF2 in Pancreatic Adenocarcinoma Cells, a Feasibility Study
Source: Biomolecules. 2026 Jun 3;16(6):828. doi: 10.3390/biom16060828 (PMC13297238; doi:10.3390/biom16060828)
Supplement: Supplementary file 1 [file biomolecules-16-00828-s001.zip › biomolecules-4289468-supplementary.pdf]

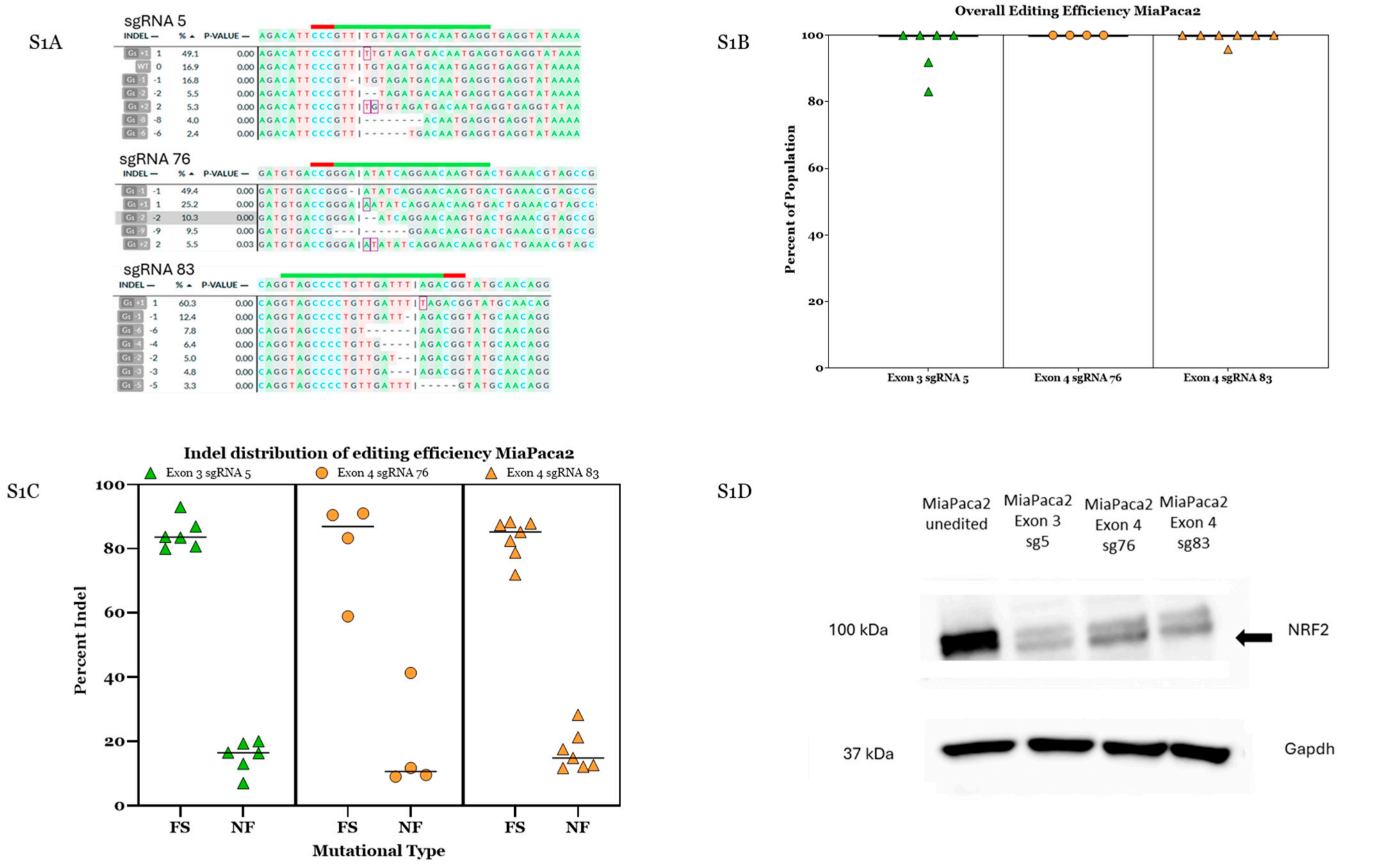

Figure S1. Genotypic and phenotypic response of NRF2 targeting in Mia-Paca-2 cells. S1A: DECODR readout of Sanger sequencing showing indel spectrum of bulk populations for sgRNA 5, 76, and 83. S1B: Overall editing efficiency of sgRNA 5, 76, and 83. S1C: Frameshift versus non-frameshift populations. S1D: Western blot showing NRF2 protein levels of unedited Mia-Paca-2 cells compared to protein levels of NRF2 in cells targeted with sgRNA 5, 76, and 83.

S2A

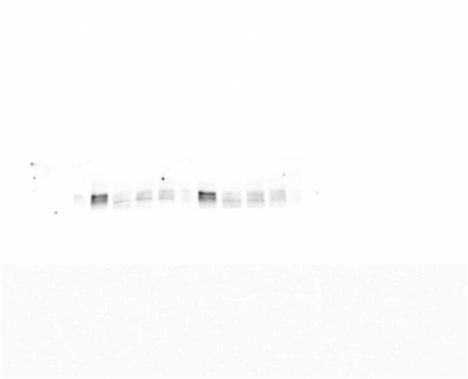

S2B

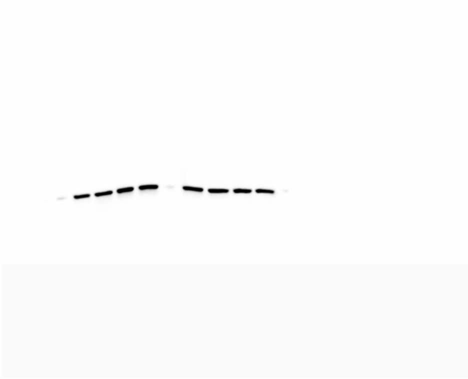

S2C

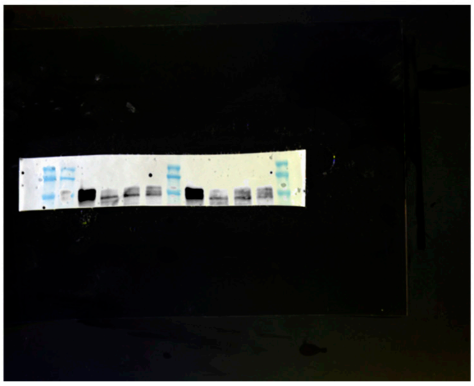

S2D

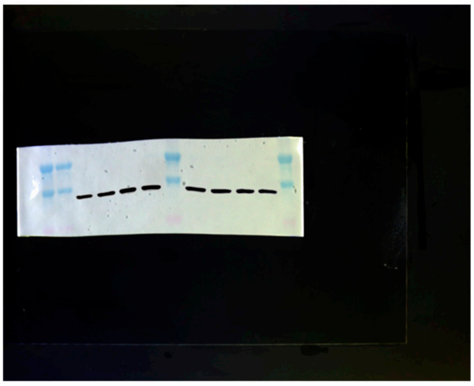

Figure S2. Original western blots PANC-1 cells. S2A: NRF2 protein levels unedited, sgRNA 5, 76, 83; 10 second exposure. S2B: Gapdh protein levels unedited, sgRNA 5, 76, 83; auto-image. S2C: NRF2 protein blot image with ladder, unedited, sgRNA 5, 76, 83; auto-image. S2D: Gapdh protein analysis image with ladder unedited, sgRNA 5, 76, 83; auto-image.

S3A

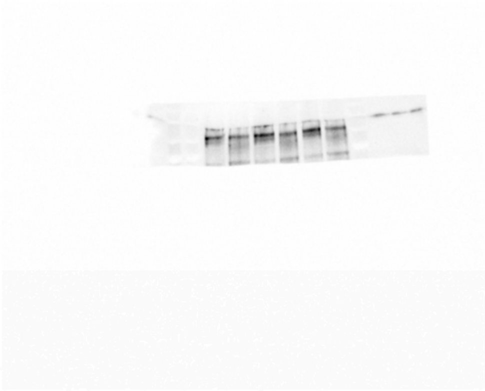

S3B

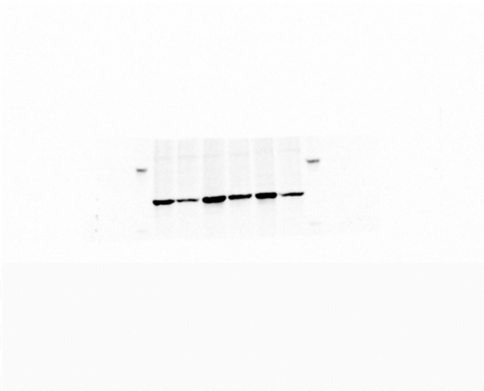

S3C

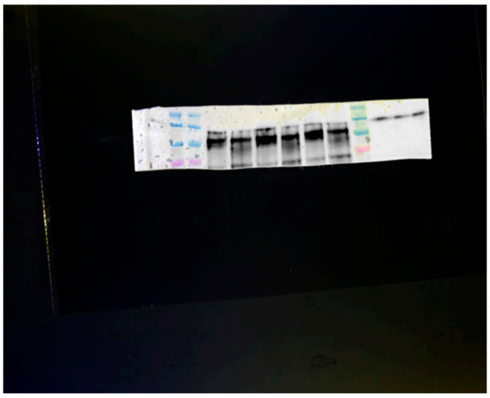

S3D

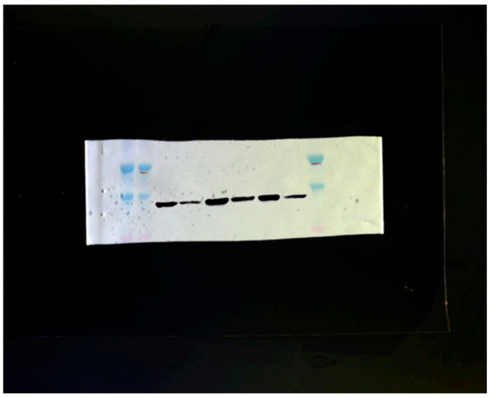

Figure S3: Original western blots PANC-1 cells. S3A: NRF2 protein levels unedited, sgRNA 3; 10 second exposure. S3B: Gapdh protein levels unedited, sgRNA 3; auto-image. S3C: NRF2 protein blot image with ladder, wildtype sgRNA 3; auto-image. S3D: Gapdh protein analysis image with ladder unedited, sgRNA 3; auto-image.

S4A

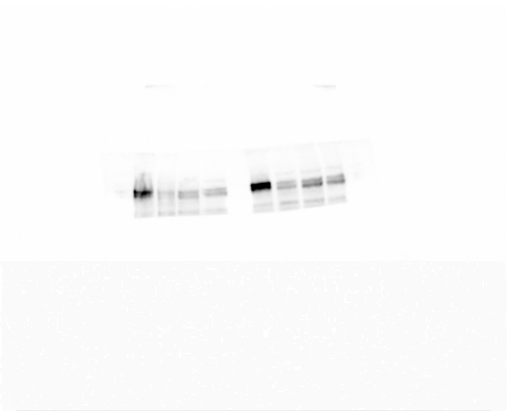

S4B

S4C

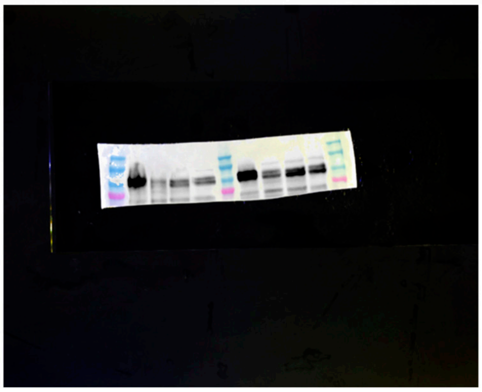

S4D

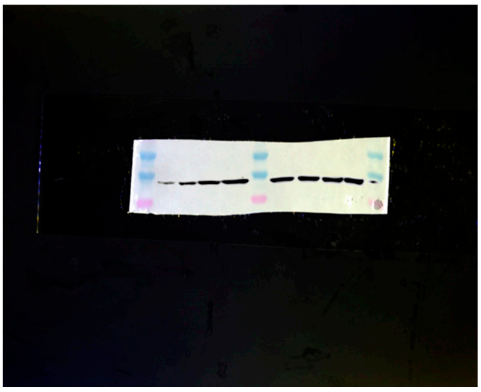

Figure S4. Original western blots Mia-Paca-2 cells. S4A: NRF2 protein blot image, wildtype sgRNA 5, 76, 83; 15 second exposure. S4B: Gapdh protein analysis image, sgRNA 5, 76, 83; auto-image. S4C: NRF2 protein blot image with ladder, wildtype, sgRNA 5, 76, 83, auto-image. S4D: Gapdh protein analysis image with ladder unedited, sgRNA 5, 76, 83; auto-image.

S5A

	Densitometry relative to Gapdh				
	Unedited	sgRNA3	sgRNA5	sgRNA76	sgRNA83
MiaPaca2	25027.86		9928.38	16709.35	6411.87
Panc1	5130.004		2039.305	3582.252	3156.09
Panc1	2346.213	1502.497			

S5B

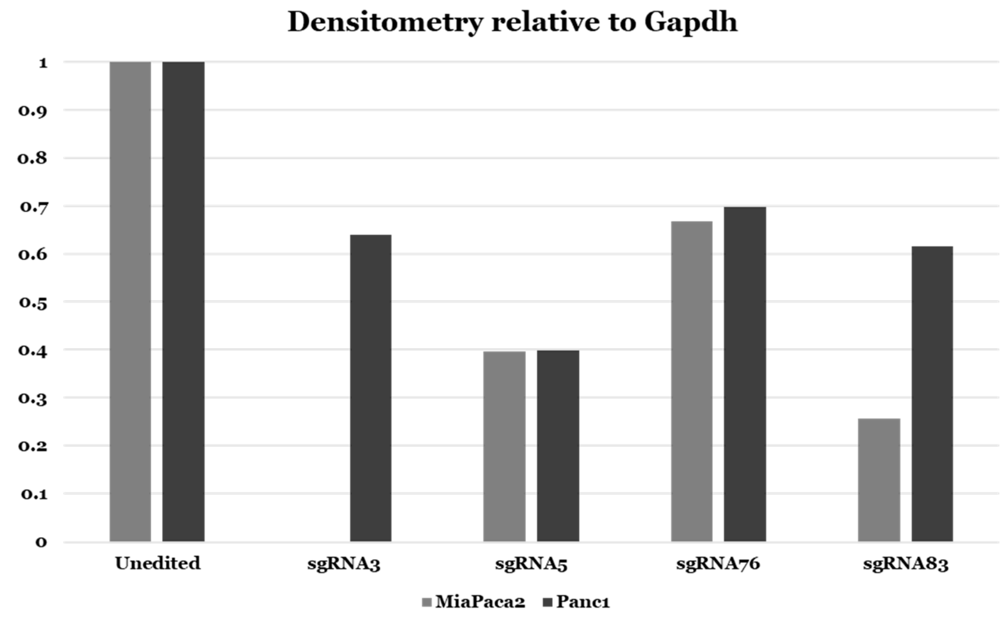

Figure S5. Densitometry calculations. S5A) Calculations to relative Gapdh for sgRNA 3, sgRNA 5, sgRNA 76, and sgRNA 83 in both Mia-Paca-2 and PANC1 cells lines. S5B) Densitometry calculations graphed, values calculated by dividing the edited population by the unedited population.
